# Comparison of clinical characteristics between adult-onset and juvenile-onset non-radiographic axial spondyloarthritis in Chinese patients: results from the COCAS cohort

**DOI:** 10.1186/s40001-023-01387-x

**Published:** 2023-09-28

**Authors:** Shu-Xin Huang, Hao-Guang Li, Hong-Jin Liang, Dan-Min Wang, Jian-Hua Peng, Feng-Cai Shen, Wei-Ping Li, Ling Lin, Zheng-Yu Xiao, Zhi-Duo Hou

**Affiliations:** 1 Department of Rheumatology and Immunology, The First Affiliated Hospital of Shantou University Medical College, Shantou, 515041 China; 2 Clinical Research Center, The First Affiliated Hospital of Shantou University Medical College, Shantou, 515041 China; 3 Department of Rheumatology and Immunology, The Second Affiliated Hospital of Nanchang University, Nanchang, 330006 China; 4 Department of Rheumatology, Shantou University Medical College, Shantou, 515041 China

**Keywords:** Non-radiographic axial spondyloarthritis, HLA-B27, Juvenile-onset, Peripheral arthritis, Enthesitis

## Abstract

**Background:**

Axial spondyloarthritis (axSpA) is a chronic inflammatory rheumatic disease predominantly affecting the axial skeleton. We aimed to describe the clinical characteristics of patients with non-radiographic axSpA (nr-axSpA) in China and compare the differences between adult- and juvenile-onset cases.

**Methods:**

A cross-sectional study was conducted using data from 776 patients with nr-axSpA in the Clinical Characteristic and Outcome in Chinese Axial Spondyloarthritis (COCAS) study cohort. Patients were divided into two groups including the adult-onset group (*n* = 662) and the juvenile-onset group (*n* = 114) according to age at disease onset. Baseline demographics and clinical characteristics were compared between patients with adult-onset and juvenile-onset nr-axSpA.

**Results:**

Overall, the male-to-female ratio was 1.26:1, the prevalence of HLA-B27 positivity was 72.2%, and the median age at disease onset of nr-axSpA was 22 years. Nearly 75% of nr-axSpA patients had peripheral arthritis in the disease course, and the prevalence of extra-articular manifestations was 10.4%. The juvenile-onset group contained a higher proportion of men (66.7% vs. 53.9%, *P* = 0.011) and a longer baseline disease duration (4.0 [4.0] vs. 1.6 [3.5], *P* < 0.001) than the adult-onset group. A family history of spondyloarthritis was more frequent in the juvenile-onset group than in the adult-onset group (23.7% vs. 15.4%, *P* = 0.028), but no significant difference in the prevalence of HLA-B27 positivity was observed between the two groups (*P* = 0.537). Regarding initial symptoms, peripheral arthritis occurred more often in patients with juvenile-onset nr-axSpA, whereas patients with adult-onset nr-axSpA presented more frequently with axial involvement. The prevalence of inflammatory back pain (IBP) was higher in the adult-onset group than in the juvenile-onset group (85.0% vs. 75.4%, *P* = 0.010), whereas the juvenile-onset group showed a higher prevalence of peripheral arthritis and enthesitis than the adult-onset group (67.5% vs. 48.5%, *P* < 0.001; 35.1% vs. 23.3%, *P* = 0.007, respectively).

**Conclusions:**

Compared with adult-onset nr-axSpA, juvenile-onset nr-axSpA was more common in men and those with a family history of spondyloarthritis. Juvenile-onset nr-axSpA presents with a “peripheral predominant” mode at disease onset and a higher frequency of peripheral arthritis and enthesitis during the disease course.

## Background

Axial spondyloarthritis (axSpA) is an inflammatory disease predominantly affecting the axial skeleton. The Assessment of SpondyloArthritis International Society (ASAS) classification criteria divides axSpA into the following two categories [1, 2]: patients with radiographic axSpA (r-axSpA), who have developed radiographic damage in the sacroiliac joint (SIJ) and fulfil the modified New York criteria (mNYc) [3], also termed ankylosing spondylitis (AS), and patients with non-radiographic axSpA (nr-axSpA) who did not meet the mNYc. However, there is currently no unified understanding of the nr-axSpA concept. One viewpoint considers that nr-axSpA and AS are homogeneous in different stages of the disease course because they share common characteristics, such as clinical presentation, clinical disease activity, treatment response, and comorbidity burden [4, 5]. However, there are nr-axSpA cases that have not progressed to AS during long-term follow-up and even evolved to other diseases, supporting the notion that nr-axSpA partially overlaps with pre-AS but is not fully equal to the early phase of AS; additionally, nr-axSpA is more clinically heterogeneous than AS [6, 7]. Although nr-axSpA is widely distributed globally, its clinical characteristics vary among ethnic and geographic groups[8]. Previous studies on nr-axSpA have focused on Caucasian populations, so the clinical characteristics of nr-axSpA in Chinese patients are not well known. Therefore, it is necessary to further study the characteristics of Chinese patients with nr-axSpA.

Several important differences in demographic features, patterns of disease onset, and clinical manifestations between adult-onset AS (AoAS) and juvenile-onset AS (JoAS) have been reported [9–11]. For example, JoAS has a higher frequency of peripheral joint involvement and a lower prevalence of HLA-B27 positivity and uveitis than AoAS. As mentioned previously, nr-axSpA and AS belong to different axSpA subgroups. However, the similarities and differences between adult- and juvenile-onset nr-axSpA remain obscure. Thus, this study aimed to summarise the clinical characteristics of Chinese patients with nr-axSpA and compare the differences between adult-onset and juvenile-onset nr-axSpA.

## Methods

This cross-sectional study used data from the Clinical Characteristic and Outcome in Chinese Axial Spondyloarthritis (COCAS; registration no. ChiCTR2100049357) cohort. The COCAS cohort has been previously described in detail [12]. The COCAS study was a single-centre ambispective cohort study initiated by the Department of Rheumatology and Immunology & Clinical Research Center in the First Affiliated Hospital of Shantou University Medical College, with the aim of evaluating the clinical characteristics and prognosis of Chinese patients with axSpA.

### Patients

**Fig. 1:**
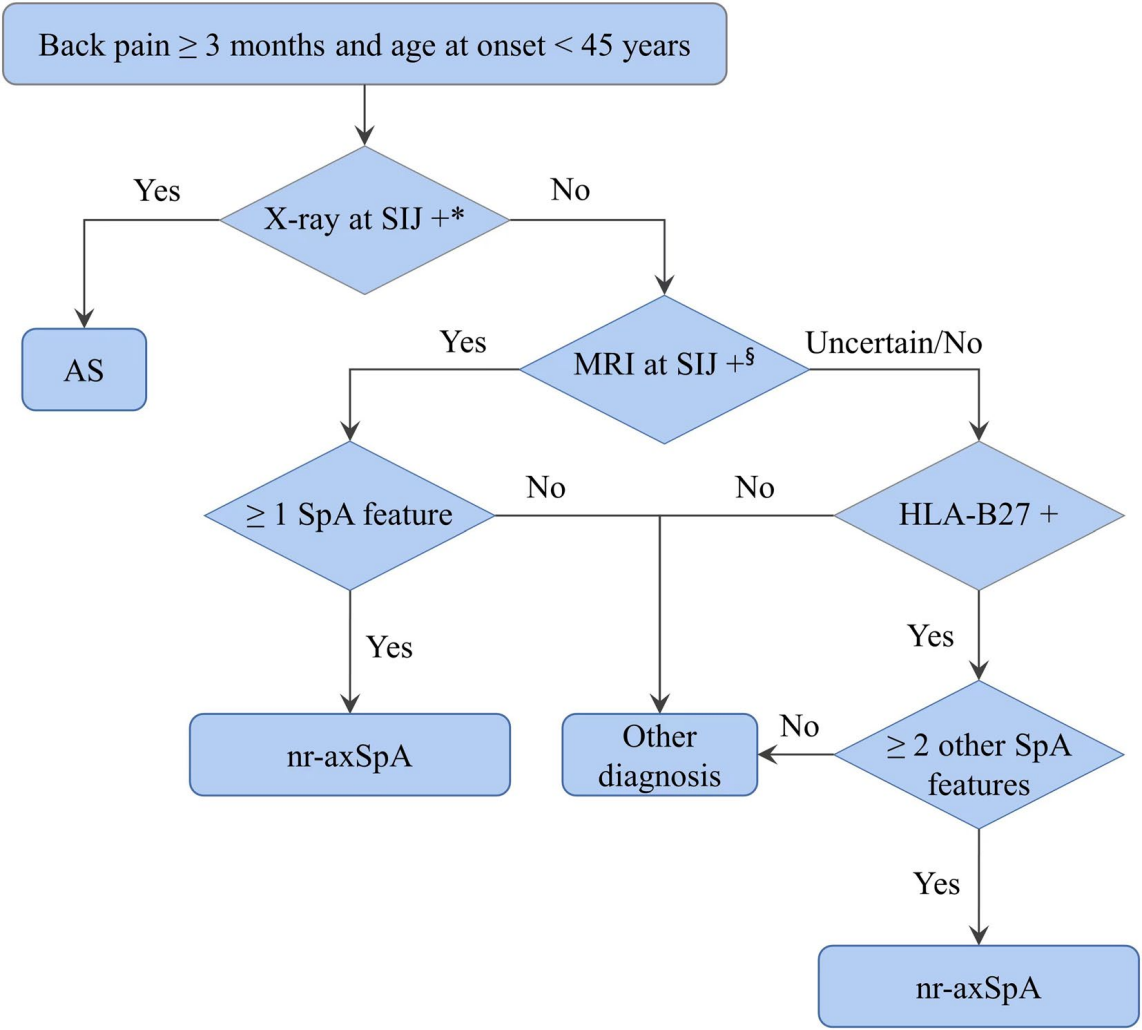
The differential diagnosis flowchart for non-radiographic axial spondyloarthritis. *X-ray criteria: ≥ grade 2 sacroiliitis bilaterally, or grade 3–4 sacroiliitis unilaterally; ^§^MRI criteria: one BME lesion on two or more consecutive slides or numerous BME lesions on one slide. SIJ, sacroiliac joint; AS, ankylosing spondylitis; MRI, magnetic resonance imaging; SpA, spondyloarthritis; HLA-B27, human leukocyte antigen B27; nr-axSpA, non-radiographic axial spondyloarthritis

A total of 776 patients who visited the Department of Rheumatology and Immunology of the First Affiliated Hospital of Shantou University Medical College from 1999 to 2020 were included in this study, including 662 patients with adult-onset nr-axSpA (age at disease onset, ≥ 16 years) and 114 with juvenile-onset nr-axSpA (age at disease onset, < 16 years). All 776 patients underwent pelvis X-ray and 656 of them underwent magnetic resonance imaging (MRI). All patients fulfilled the ASAS classification criteria for axSpA [1, 2] but did not meet the mNYc [3]. The differential diagnosis flowchart for nr-axSpA is shown in Fig. 1.

This study was approved by the Ethics Committee of the First Affiliated Hospital of Shantou University Medical College. Informed consent was obtained from all study participants.

### Data collection

Recorded clinical data included sex, age at first visit, age at disease onset, baseline disease duration, human leukocyte antigen (HLA)-B27 status, family history of SpA (presence of ankylosing spondylitis, acute anterior uveitis, psoriasis, inflammatory bowel disease, or reactive arthritis in first- or second-degree relatives), clinical manifestations, C-reactive protein (CRP) level (elevated CRP was defined as > 8 mg/L), erythrocyte sedimentation rate (ESR) level (elevated ESR was defined as > 15 mm/h and > 20 mm/h in male and female patients, respectively), Ankylosing Spondylitis Disease Activity Score (ASDAS) (high disease activity was defined as > 2.1), SIJ imaging assessment (MRI and plain radiography) at the time of study inclusion (baseline), and medication use. The images were assessed independently by a trained rheumatologist and a radiologist. In cases of disagreement, the final decision on the presence or absence of findings was made by another experienced rheumatologist. Bone marrow oedema (BME) lesions that are strongly indicative of SpA (one BME lesion on two or more consecutive slides or numerous BME lesions on one slide) were considered SIJ-MRI positivity [13]. The MRI protocol for evaluating the sacroiliac joints consisted of oblique coronal T1, T2 (without fat suppression), and short tau inversion recovery (STIR) sequences oriented parallel to the longitudinal axis of the sacrum. Radiographic sacroiliitis was graded based on the mNYc (1984) [3]. Enthesitis was defined as tenderness at the insertion point of a ligament or tendon into the bone on palpation [14]. Inflammatory back pain (IBP) was defined using the ASAS criteria [15].

### Statistical analysis

All data analyses were performed using SPSS for Windows version 26.0 (IBM Corp., Armonk, NY, USA). The Kolmogorov–Smirnov test of normality was performed for continuous data. Normally distributed variables are presented as mean with standard deviations (SDs), while non-normally distributed variables are presented as medians with interquartile ranges (IQRs). Independent t-tests were performed for normally distributed continuous variables and the Mann–Whitney U test for non-normally distributed variables. For statistical comparison of categorical variables, such as proportions, the Chi-square test or Fisher’s exact test was performed. Statistical significance was assigned at *P* < 0.05.

## Results

### Demographic and clinical characteristics

Table 1 shows the baseline demographic and clinical characteristics of 776 patients with nr-axSpA. Male patients had a significantly earlier age at disease onset and age at diagnosis than female patients (19.0 [10.0] vs. 24.0 [10.0], *P* < 0.001; 22.0 [12.0] vs. 27.0 [12.0], *P* < 0.001, respectively). The prevalence of HLA-B27 positivity was more frequent in male patients than in female patients (78.8% vs. 63.8%, *P* < 0.001).

**Table 1:** Baseline demographics and clinical characteristics of 776 cases of non-radiographic axial spondyloarthritis*

Parameter		Total (*n* = 776)	Male (*n* = 433)	Female (*n* = 343)	*P*†
Age at disease onset, (yrs), Median (IQR)		22.0 (11.0)	19.0 (10.0)	24.0 (10.0)	< **0.001**^#^
Age at first visit, (yrs), Median (IQR)		24.0 (13.0)	22.0 (12.0)	27.0 (12.0)	< **0.001**^#^
Baseline disease duration, (yrs), Median (IQR)		2.0 (3.5)	2.0 (3.5)	2.0 (4.5)	0.574^#^
HLA-B27 positive		560 (72.2)	341 (78.8)	219 (63.8)	< **0.001**
Family history of SpA		129 (16.6)	76 (17.6)	53 (15.5)	0.435
Initial symptoms	Axial	435 (56.1)	234 (54.0)	201 (58.6)	0.204
	Peripheral	326 (42.0)	190 (43.9)	136 (39.7)	0.236
	Extra-articular	16 (2.1)	9 (2.1)	7 (2.0)	0.971
Symptom, ever	Axial only	194 (25.0)	98 (22.6)	96 (28.0)	0.087
	Peripheral	582 (75.0)	335 (77.4)	247 (72.0)	0.087
	Lower limbs	398 (51.3)	228 (52.7)	170 (49.6)	0.392
	Upper limbs	167 (21.5)	81 (18.7)	86 (25.1)	**0.032**
	Extra-articular	81 (10.4)	43 (9.9)	38 (11.1)	0.603
Spinal pain (baseline), 0–10 NRS, Median (IQR)^‡^		5.0 (2.0)	5.0 (2.0)	5.0 (2.0)	0.488
Patient’s global assessment (baseline), 0–10 NRS, Median (IQR)^‡^		6.0 (2.0)	6.0 (2.0)	6.0 (2.0)	**0.002**
Pain/swelling peripheral joints (baseline), 0–10 NRS, Median (IQR)^‡^		3.0 (5.0)	3.0 (6.0)	3.0 (5.0)	0.116
Duration of morning stiffness (baseline), 0–10 NRS, Median (IQR)^‡^		1.0 (3.0)	2.0 (3.0)	1.0 (3.0)	0.140
Elevated ESR (baseline)		328 (42.3)	209 (48.3)	119 (34.7)	< **0.001**
Elevated CRP (baseline)		314 (40.5)	213 (49.2)	101 (29.4)	< **0.001**
ASDAS-ESR (baseline), Median (IQR)^‡^		2.7 (1.3)	2.7 (1.4)	2.6 (1.2)	0.170^#^
ASDAS-CRP (baseline), Median (IQR)^‡^		2.7 (1.3)	3.0 (1.5)	2.4 (1.1)	< **0.001**^#^
ASDAS-ESR (baseline) > 2.1^‡^		372 (74.3)	211 (74.8)	161 (73.5)	0.740
ASDAS-CRP (baseline) > 2.1^‡^		377 (75.2)	225 (80.1)	152 (69.1)	**0.005**
SIJ-MRI positivity, *n* (%)^§^		383 (58.4)	206 (56.9)	177 (60.2)	0.394
Medications					
NSAIDs monotherapy		382 (49.2)	206 (47.6)	176 (51.3)	0.301
NSAIDs + csDMARDs		313 (40.3)	167 (38.6)	146 (42.6)	0.260
NSAIDs + bDMARDs		81 (10.4)	60 (13.9)	21 (6.1)	** < 0.001**

HLA-B27 = human leukocyte antigen B27; SpA = spondyloarthritis; NRS = numerical rating scale; ASDAS-CRP = Ankylosing Spondylitis Disease Activity Score; CRP = C-reactive protein; ESR = erythrocyte sedimentation rate; MRI-SIJ = MRI of the sacroiliac joints; NSAIDs = non-steroidal anti-inflammatory drugs; csDMARDs = conventional synthetic disease-modifying anti-rheumatic drugs; bDMARDs = biological disease-modifying anti-rheumatic drugs

Bold values indicate statistical significance with *P* value less than 0.05

^*^Values are the n (%) unless otherwise indicated. † Chi-square test was used to compare male and female patients unless otherwise indicated

^#^Mann–Whitney U test

^‡^Available in 501 patients (*n* = 281 for male and *n* = 220 for female)

^§^Available in 656 patients (*n* = 362 for male and *n* = 294 for female)

Axial symptoms occurred in more than half (56.1%) of the nr-axSpA patients at disease onset. During the disease course, a great proportion of these patients (75.0%) presented with both axial and peripheral symptoms. Extra-articular manifestations, including inflammatory bowel disease, psoriasis, and uveitis, were observed in 10.4% of the patients.

The proportion of cases with elevated CRP levels and ESRs at baseline was 40.5% and 42.3%, respectively. The median ASDAS-CRP was 2.7 (1.3), and nearly 75% of the patients had high disease activity (ASDAS > 2.1). At baseline, the proportion of male patients with increased CRP levels and ESRs was higher than that of female patients (49.2% vs. 29.4%, *P* < 0.001; 48.3% vs 34.7%, *P* < 0.001, respectively). Male patients also had a greater prevalence of high disease activity (ASDAS-CRP > 2.1; 80.1% vs. 69.1%, *P* = 0.005) and higher median ASDAS-CRP (3.0 [1.5] vs. 2.4 [1.1], *P* < 0.001) than female patients. There were 383 (49.4%) patients who showed active sacroiliitis with BME lesions on MRI. They were classified as nr-axSpA using the “imaging arm” of the ASAS criteria. The remaining 273 (35.2%) who did not show active sacroiliitis on MRI and the other 120 (15.5%) whose MRIs were unavailable could only be classified as having nr-axSpA using the “clinical arm” of the ASAS classification criteria. No significant difference in the proportion of SIJ-MRI positivity was observed between the sexes (*P* > 0.05).

All patients were administered medication, including non-steroidal anti-inflammatory drugs (100%), conventional synthetic disease-modifying anti-rheumatic drugs (40.3%), and biological disease-modifying anti-rheumatic drugs (10.4%). However, it is worth noting that only 24% of the patients received exercise instruction.

### Comparison of adult-onset versus juvenile-onset nr-axSpA

Table 2 shows a comparison of demographic and clinical characteristics between patients with adult-onset and juvenile-onset nr-axSpA. Compared with the adult-onset nr-axSpA group, the juvenile-onset nr-axSpA group had a higher proportion of male patients (66.7% vs. 53.9%, *P* = 0.011) and a longer baseline disease duration (4.0 [4.0] vs. 1.6 [3.5], *P* < 0.001). A family history of SpA was more frequent in juvenile-onset nr-axSpA than in adult-onset nr-axSpA (23.7% vs. 15.4%, *P* = 0.028), but no significant difference in the prevalence of HLA-B27 positivity was observed between the two groups. Regarding the initial symptoms, peripheral arthritis occurred more often in patients with juvenile-onset nr-axSpA, whereas patients with adult-onset nr-axSpA presented more frequently with axial involvement. During the disease course, the prevalence of peripheral arthritis was also higher in juvenile-onset nr-axSpA than in adult-onset nr-axSpA (83.3% vs. 73.6%, *P* = 0.026). No significant difference was found in inflammatory markers (ESR/CRP), disease activity (ASDAS), SIJ-MRI positivity, or medication use between the two groups (*P* > 0.05). The grades of radiographic changes in the SIJs of the adult-onset group were observed equally with the juvenile-onset group, with the exception of bilateral grade 0, which was observed less frequently in the juvenile-onset group.

**Table 2:** Comparisons of baseline demographics and clinical characteristics between adult-onset and juvenile-onset non-radiographic axial spondyloarthritis*

Parameter		Adult onset (*n* = 662)	Juvenile onset (*n* = 114)	*P*†
Male		357 (53.9)	76 (66.7)	**0.011**
Age at disease onset, (yrs), Median (IQR)		23.0 (10.0)	13.5 (3.0)	< **0.001**^#^
Age at first visit, (yrs), Median (IQR)		25.0 (12.0)	17.0 (2.3)	< **0.001**^#^
Baseline disease duration, (yrs), Median (IQR)		1.6 (3.5)	4.0 (4.0)	< **0.001**^#^
HLA-B27 positive		475 (71.8)	85 (74.6)	0.537
Family history of SpA		102 (15.4)	27 (23.7)	**0.028**
Initial symptoms	Axial	392 (59.2)	43 (37.7)	< **0.001**
	Peripheral	257 (38.8)	69 (60.5)	< **0.001**
	Extra-articular	14 (2.1)	2 (1.8)	1.000
Symptom, ever	Axial only	175 (26.4)	19 (16.7)	**0.026**
	Peripheral	487 (73.6)	95 (83.3)	**0.026**
	Lower limbs	321 (48.5)	77 (67.5)	< **0.001**
	Upper limbs	144 (21.8)	23 (20.2)	0.705
	Extra-articular	66 (10.0)	15 (13.2)	0.304
Spinal pain (baseline), 0–10 NRS, Median (IQR)^‡^		5.0 (2.0)	5.0 (3.0)	0.058^#^
Patient’s global assessment (baseline), 0–10 NRS, Median (IQR)^‡^		6.0 (2.0)	6.0 (2.0)	0.692^#^
Pain/swelling peripheral joints (baseline), 0–10 NRS, Median (IQR)^‡^		2.5 (5.0)	5.0 (6.0)	**0.012**^#^
Duration of morning stiffness (baseline), 0–10 NRS, Median (IQR)^‡^		1.0 (3.0)	1.0 (3.0)	0.675^#^
Elevated ESR (baseline)		278 (42.0)	50 (43.9)	0.710
Elevated CRP (baseline)		260 (39.3)	54 (47.4)	0.104
ASDAS-ESR (baseline), Median (IQR)^‡^		2.7 (1.3)	2.7 (1.3)	0.883^#^
ASDAS-CRP (baseline), Median (IQR)^‡^		2.7 (1.3)	2.8 (1.3)	0.932^#^
ASDAS-ESR (baseline) > 2.1^‡^		313 (74.2)	59 (74.7)	0.924
ASDAS-CRP (baseline) > 2.1^‡^		318 (75.4)	59 (74.7)	0.899
Sacroiliac joint radiographic findings				
Bilateral grade 0		308 (46.5)	41 (36.0)	**0.036**
Unilateral grade 1		127 (19.2)	24 (21.1)	0.642
Unilateral grade 2		72 (10.9)	15 (13.2)	0.476
Bilateral grade 1		118 (17.8)	26 (22.8)	0.206
Combined, unilateral grades 1 and 2		37 (5.6)	8 (7.0)	0.547
SIJ-MRI positivity^§^		328 (58.9)	55 (55.6)	0.535
Bilateral BME lesions		198 (60.4)	27 (49.1)	0.116
Unilateral BME lesions		130 (39.6)	28 (50.9)	
Medications				
NSAIDs monotherapy		326 (49.2)	56 (49.1)	0.981
NSAIDs + csDMARDs		264 (39.9)	49 (43.0)	0.533
NSAIDs + bDMARDs		72 (10.9)	9 (7.9)	0.336

HLA-B27 = human leukocyte antigen B27; SpA = spondyloarthritis; NRS = numerical rating scale; ASDAS = Ankylosing Spondylitis Disease Activity Score; ESR = erythrocyte sedimentation rate; CRP = C-reactive protein; SIJ-MRI = sacroiliac joint-magnetic resonance imaging; BME = Bone marrow oedema; NSAIDs = non-steroidal anti-inflammatory drugs; csDMARDs = conventional synthetic disease-modifying anti-rheumatic drugs; bDMARDs = biological disease-modifying anti-rheumatic drugs

Bold values indicate statistical significance with *P* value less than 0.05

^*^Values are the n (%) unless otherwise indicated. † Chi-square test was used to compare adult-onset and juvenile-onset groups unless otherwise indicated

^#^Mann–Whitney U test

^‡^Available in 501 patients (*n* = 79 for juvenile-onset group and *n* = 422 for adult-onset group)

^§^Available in 656 patients (*n* = 99 for the juvenile-onset group and *n* = 557 for adult-onset group)

**Fig. 2:**
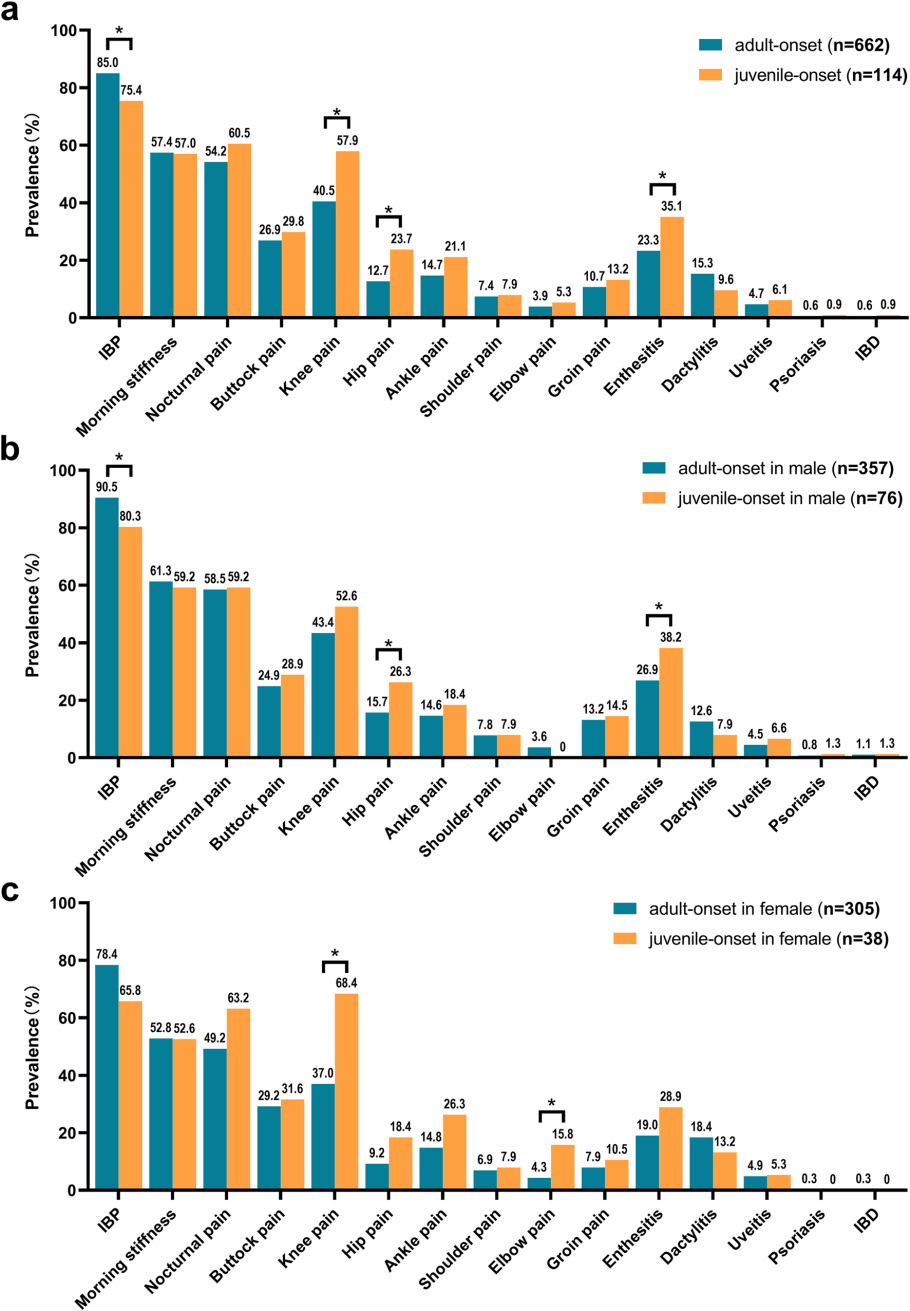
Comparison of clinical manifestations between adult-onset and juvenile-onset non-radiographic axial spondyloarthritis. **a**. adult onset vs. juvenile onset. Inflammatory back pain occurred more often in adult-onset group than in juvenile-onset group (85.0% vs. 75.4%, *P* = 0.010), while hip pain, knee pain, and enthesitis occurred more often in juvenile-onset group than in adult-onset group (23.7% vs. 12.7%, *P* = 0.002; 57.9% vs. 40.5%, *P* = 0.001; 35.1% vs. 23.3%, *P* = 0.007, respectively); **b**. adult onset in male vs. juvenile onset in male. Inflammatory back pain occurred more often in adult-onset nr-axSpA than in juvenile-onset nr-axSpA in male (90.5% vs. 80.3%, *P* = 0.011), while hip pain, and enthesitis occurred more often in juvenile-onset nr-axSpA than in adult-onset nr-axSpA in male (26.3% vs. 15.7%, *P* = 0.027; 38.2% vs. 26.9%, *P* = 0.049, respectively); **c**. adult onset in female vs. juvenile onset in female. Elbow pain and knee pain occurred more often in juvenile-onset nr-axSpA than in adult-onset nr-axSpA in female (15.8% vs. 4.3%, *P* = 0.011; 68.4% vs. 37.0%, *P* < 0.001, respectively). IBP, inflammatory back pain; IBD, inflammatory bowel disease; *Statistically significant difference (*P* < 0.05)

Figure 2 shows a comparison of the clinical manifestations between adult-onset and juvenile-onset nr-axSpA. The prevalence of IBP was higher in the adult-onset group than in the juvenile-onset nr-axSpA group (85.0% vs. 75.4%, *P* = 0.010), and a significant difference was recorded mainly in male patients (90.5% vs. 80.3%, *P* = 0.011). Patients with juvenile-onset nr-axSpA showed a significantly higher prevalence of knee and hip involvement than patients with adult-onset nr-axSpA (57.9% vs. 40.5%, *P* = 0.001; 23.7% vs. 12.7%, *P* = 0.002, respectively). The difference in the incidence of hip involvement occurred mainly in the male group (26.3% vs. 15.7%, *P* = 0.027), whereas the difference in the incidence of knee involvement occurred mainly in the female group (68.4% vs. 37.0%, *P* < 0.001). Enthesitis was more common in juvenile-onset nr-axSpA than in adult-onset nr-axSpA (35.1% vs. 23.3%, *P* = 0.007), with the difference being significant in men but not in women (38.2% vs. 26.9%; *P* = 0.049 vs. *P* > 0.05).

## Discussion

In this study, we summarised the demographic and clinical characteristics of nr-axSpA in China and compared the differences between adult-onset and juvenile-onset nr-axSpA for the first time, using a current study on a large sample size of patients with nr-axSpA in China.

In this study, the median age at disease onset of nr-axSpA was 22 years, with a median of 23 years in the adult-onset group, similar to the results reported in the Korean and Indian cohorts (26.4 and 24.8 years, respectively) [16, 17]. By contrast, the median of this study was lower than that in Western cohorts, in which the mean ages at disease onset were > 30 years [4, 18], suggesting that the disease may develop at an earlier age in Asian populations than in Western populations [8].

In our study, the prevalence of HLA-B27 positivity was 72.2% in patients with nr-axSpA. To date, there has been a lack of large-scale epidemiological survey results on the prevalence of HLA-B27 positivity among Chinese patients with nr-axSpA. A previous study reported that more than 90% of AS patients in China were HLA-B27 positive [19], which was significantly higher than the results of our study, indicating that the rate of HLA-B27 positivity in nr-axSpA was lower than that of AS in China.

Sex has consistently been identified as a major demographic difference in nr-axSpA compared with AS, usually exhibiting a comparable sex ratio [6]. In this study, the male-to-female ratio in nr-axSpA was 1.26:1, which is lower than that in AS, but higher than that in Western cohorts of nr-axSpA such as DESIR (0.89:1) [18], SPACE (0.82:1) [18], and SCQM (0.88:1) [20]. This may reflect regional differences in the demographic characteristics of nr-axSpA patients, as in other studies of Asian nr-axSpA population, a higher proportion of male was also observed than that in Western nr-axSpA population [8, 21], suggesting that the proportion of male in nr-axSpA in Asian population may be higher than that in Western population. However, it is important to point out that the male-to-female ratio in nr-axSpA is much lower than that in AS for the same race, no matter in Asian population or European population. In addition, several differences exist in the demographic characteristics between sexes. For example, male patients had a younger age at disease onset and diagnosis and a higher prevalence of HLA-B27 positivity than female patients. A similar finding was reported in a Swiss Clinical Quality Management cohort study [22]. It is supposed that genotypic differences between sexes may be a reason for these demographic differences.

IBP is a key symptom of axial involvement and is present in most patients with axSpA. The total prevalence of IBP in our study was 83.6%, which is consistent with a large meta-analysis reporting a prevalence ranging from 71.5% to 95.4% [23]. In addition, nearly 75% of patients with nr-axSpA had peripheral involvement during the disease course. Extra-articular manifestations occurred in 10.4% of patients, and uveitis is considered one of the most common extra-articular manifestations; 4.9% of patients experienced uveitis in our study, much lower than that reported in Indian and Western cohorts (8%–12.4%) [4, 16, 18]. This finding can be partly explained by the varying prevalence of uveitis among ethnicities and geographic regions. A previous study reported that the prevalence of uveitis in Chinese patients with AS was much lower than that reported in Western, Korean, and Indian studies [21, 24]. Another explanation might be the shorter disease duration of patients with nr-axSpA in our study, which is supported by the viewpoint that uveitis in axSpA does not always occur in the early stage of the disease and requires time to develop [25, 26].

Our data showed that most patients had high disease activity (ASDAS > 2.1) at baseline; however, there were differences between sexes. The proportions of male patients with ASDAS-CRP > 2.1 and elevated CRP levels and ESRs were higher than those of female patients. Additionally, the median ASDAS-CRP level was higher in male patients than female patients. The conclusion that acute-phase reactants and ASDAS are important positive predictors of radiographic sacroiliitis progression in axSpA has been reported in a previous cohort study [27]. Whether male patients are more likely to experience progression of structural damage than female patients should be further investigated through long-term cohort studies with larger sample sizes.

In the realm of treatment, the importance of exercise is on par with pharmacological interventions. However, our study revealed that only a small percentage of patients with nr-axSpA received exercise instruction. Research indicates that when applying attentional focus strategies, an External Focus of Attention (EFA) seems to be more effective than an Internal Focus of Attention (IFA) in affecting the movement execution in patients with musculoskeletal disorders [28]. Although there is limited research comparing the effectiveness of EFA and IFA guidance in axSpA patients, there is a need for further exploration to determine the appropriate rehabilitative strategies and motor learning methodologies for this population.

Several important differences in the demographic features, patterns of disease onset, and clinical manifestations have been reported between JoAS and AoAS. In this study, we also compared adult-onset and juvenile-onset nr-axSpA. Several significant differences were identified between the two groups regarding the demographic characteristics (Table 2). First, our study revealed that there were significantly more male patients in the juvenile-onset group than in the adult-onset group. Male predominance has also been reported in previous studies on juvenile spondyloarthritis (JSpA), enthesitis-related JIA (ERA), and other subtypes of juvenile idiopathic arthritis (JIA), findings which align with our study. Previous studies have reported that male patients were associated with worse functional outcomes and poorer prognosis and thus seek health care more frequently than female patients, which also contributes to the phenomenon (male bias) [29, 30]. Second, the rate of family history of SpA was slightly higher in the juvenile-onset group than in the adult-onset group; however, no significant difference in HLA-B27 status was identified between the two groups, indicating that genetic factors other than HLA-B27 may play a role in disease pathogenesis. In addition, our data demonstrated that the disease duration was longer in juvenile-onset nr-axSpA than in adult-onset nr-axSpA, indicating that a longer diagnostic delay may occur more frequently in the juvenile-onset group. Currently, no standard classification system exists for juvenile-onset axSpA. Adult classification criteria such as Amor and the European Spondyloarthropathy Study Group have been more often applied to these patients in previous studies [31, 32]. In paediatric rheumatology, these patients may be categorised as JIA, as defined by the International League of Associations for Rheumatology classification criteria [33]. The application of this classification system in clinical practice aids in identifying patients with similar clinical and prognostic implications. However, sensitive imaging tools, such as MRI, which have contributed to a better assessment of patients with early disease stages of axSpA are not included in these classification systems. Our previous report confirmed that MRI can predict the progression of juvenile-onset nr-axSpA to JoAS [12]. However, refining definitions of “positive” MRI in juvenile-onset nr-axSpA is required in future studies [34].

Several similarities were observed in the clinical manifestations between the two groups. For example, IBP, one of the key clinical manifestations, occurred in the majority of patients in both groups. In addition, morning stiffness, nocturnal pain, buttock pain, groin pain, and extra-articular manifestation were equally frequent between the two groups. However, the axial/peripheral pattern of disease at onset differed significantly; the juvenile-onset group experienced a more frequent onset of peripheral joint involvement, whereas axial involvement was not as frequent as that in the adult-onset group (Table 2). The disease-onset pattern of juvenile-onset nr-axSpA may present as a mode of “peripheral predominant”. A similar conclusion was also demonstrated in an earlier cross-sectional study comparing JoAS and AoAS [35–37]. Therefore, typical axial skeleton involvement should not be expected as the first clinical presentation for patients with juvenile-onset nr-axSpA. Furthermore, this clinical feature of “peripheral predominant” continued in the disease course, especially knee and hip involvement (Fig. 1), which is thought to be associated with a poor functional outcome. This finding has important therapeutic implications, as juvenile-onset nr-axSpA patients may require closer monitoring and more aggressive treatment for hip disease. In addition, enthesitis is more common in juvenile-onset nr-axSpA. Enthesitis is a factor for which the progression to JoAS was suggested in our previous study [12]. Furthermore, enthesitis was found to be a risk factor for sacroiliitis in an Italian cohort study on ERA [38].

The ASDAS was used as a clinical tool for measuring disease activity among the patients in our study. Although the level of disease activity was highly comparable between the two groups, juvenile-onset nr-axSpA had more severe disease effects in terms of peripheral pain/swelling measured by a numerical rating scale (Table 2). Moreover, the ASDAS did not include the measure domain of enthesitis. Hence, the ASDAS may fail to assess accurately the disease activity of juvenile-onset patients. Future research might require the exploration of better assessment tools for measuring disease activity in juvenile-onset nr-axSpA while increasing the weight of key stakeholders, such as peripheral arthritis and enthesitis.

Our study had several strengths, particularly the inclusion of a large number of patients from a single centre, thereby reducing the possible effects of population heterogeneity. However, this study also had some limitations. First, as this study used retrospectively collected data from the COCAS cohort, some patient information was lacking (including ASDAS score and SIJ-MRI). Second, although juvenile-onset nr-axSpA was used to define patients who experienced symptom onset at age < 16 years, all patients enrolled in the current study were aged ≥ 16 years at their first visit to the adult Department of Rheumatology. Thus, these patients were not fully representative of the juvenile-onset nr-axSpA population, especially those still in childhood. Third, no comparison between juvenile-onset and adult-onset groups related to functional outcomes was performed in this study, and further studies including a functional assessment and prognosis analysis of nr-axSpA are warranted.

## Conclusions

Compared with adult-onset nr-axSpA, juvenile-onset nr-axSpA was more common in male patients and was correlated with a positive family history of SpA. Juvenile-onset nr-axSpA presents with a “peripheral predominant” mode at disease onset and a higher frequency of peripheral arthritis and enthesitis during the disease course. Future studies including larger populations and longer-term follow-up, with the aim of evaluating clinical characteristics and prognosis, are desirable.

## Data Availability

The datasets used during the current study are available from the corresponding author on reasonable request.

## Abbreviations

axSpA: Axial spondyloarthritis
SIJ: Sacroiliac joint
ASAS: Assessment of SpondyloArthritis International Society
r-axSpA: Radiographic axSpA
AS: Ankylosing spondylitis
mNYc: Modified New York criteria
nr-axSpA: Non-radiographic axSpA
AoAS: Adult-onset AS
JoAS: Juvenile-onset AS
COCAS: Clinical Characteristic and Outcome in Chinese Axial Spondyloarthritis
SpA: Spondyloarthritis
HLA: Human leukocyte antigen
ESR: Erythrocyte sedimentation rate
CRP: C-reactive protein
ASDAS: Ankylosing spondylitis disease activity score
MRI: Magnetic resonance imaging
BME: Bone marrow oedema
IBP: Inflammatory back pain
SD: Standard deviation
IQR: Interquartile range
JSpA: Juvenile spondyloarthritis
ERA: Enthesitis-related JIA
JIA: Juvenile idiopathic arthritis
